# Effects of two endoscopic posterior lumbar interbody fusion surgical sequences for treatment of unstable lumbar spinal stenosis: a retrospective study

**DOI:** 10.3389/fsurg.2025.1631903

**Published:** 2025-09-05

**Authors:** Hengyan Pan, Ruibing Feng, Xiaofeng Duan, Yizheng Huang, Hao Hu, Gang Wu, Yong Huang

**Affiliations:** Department of Spine Surgery, Hubei Provincial Hospital of Traditional Chinese Medicine, Wuhan, Hubei, China

**Keywords:** endoscopic posterior lumbar interbody fusion, surgical sequence, lumbar spinal stenosis, spinal fusion, minimally invasive surgical procedures

## Abstract

**Background:**

Endoscopic posterior lumbar interbody fusion (Endo-PLIF) is commonly employed for the treatment of unstable lumbar spinal stenosis (ULSS). However, the impact of different surgical sequences on clinical outcomes remains unclear. This study aims to compare outcomes between two Endo-PLIF surgical sequences for ULSS.

**Method:**

This retrospective study analyzed ULSS patients who underwent Endo-PLIF at our institution from 2019 to 2023. Group A had guidewire placement before decompression, while Group B had it after. Both groups began with localization and concluded with percutaneous screw fixation. Primary outcomes measured were operative time, fluoroscopy frequency, pedicle screw accuracy (using Gertzbein and Robbins criteria on postoperative CT), and functional scores (VAS, ODI). Fusion rates and complications were also evaluated.

**Result:**

Group A demonstrated significantly shorter operative time and fewer intraoperative fluoroscopies than Group B. Furthermore, pedicle screw placement exhibited a higher rate of excellent/good grades (Gertzbein and Robbins) in Group A. Both groups achieved significant and equivalent improvements in VAS and ODI scores at 1-year follow-up compared to preoperative baselines. Fusion rates and overall complication rates did not differ significantly between the groups.

**Conclusions:**

For patients with ULSS, following the surgical procedure of inserting the guide wire before decompression can lead to more satisfactory clinical outcomes.

## Introduction

Lumbar spinal stenosis represents a common indication for spinal surgery among patients aged over 65 years. Projections suggest that by 2025, the prevalence of lumbar spinal stenosis within the elderly demographic will rise by 59%, potentially affecting 640 million individuals (1–4).With an aging population and the increasing desire of patients to maintain mobility in later life, improving the quality of life for those with lumbar spinal stenosis has emerged as a critical global issue (5, 6).

Surgical intervention is preferred when conservative treatments fail, as their mechanisms for symptom relief of lumbar spine stenosis remain unclear (7).With the maturation of spinal endoscopy technology and the development of novel instruments, endoscopic decompression and fusion have become the primary surgical approach for unstable lumbar spinal stenosis (ULSS). This procedure is specifically referred to as endoscopic posterior lumbar interbody fusion (Endo-PLIF) (8, 9).

This minimally invasive decompression technique maintains the integrity of the posterior lumbar structural elements and reduces collateral damage. Its primary advantage is the prevention of iatrogenic instability at the surgical segment, a complication commonly associated with open surgical techniques (10–14). Nevertheless, this procedure has not been widely adopted due to its steep learning curve, long operating time, and higher complication rates. Current research initiatives are concentrated on optimizing the surgical workflow, decreasing operative time, reducing fluoroscopy time, enhancing the accuracy of screw placement, and identifying the most effective surgical sequence to promote the wider implementation of this procedure (15). Currently there is no standardized surgical workflow for this procedure in clinical practice. Two commonly reported sequences are (1) C-arm x-ray-assisted localization and marking of the puncture point, guide wire insertion, sufficient decompression and bone graft fusion, percutaneous screw placement and internal fixation, and (2) C-arm x-ray-assisted localization and marking of the puncture point, sufficient decompression and bone graft fusion, guidewire insertion, percutaneous screw placement and internal fixation. Currently, there is a deficiency of comparative studies examining these sequences.

In this study, we conducted a retrospective analysis of clinical data from 143 cases of Endo-PLIF performed by our minimally invasive spine surgery team for the treatment of ULSS between October 2019 and June 2023. The primary objective was to compare the clinical outcomes between these two surgical sequences.

## Methods and materials

### Patient population

This study received approval from the ethical committee of Hubei Provincial Hospital of Traditional Chinese Medicine (Ethics Reference No: HBZY2022-C03-02), and written informed consent was obtained from all participants prior to the provision of detailed procedural explanations. Standard perioperative and postoperative outcome data were systematically collected as part of the Hospital Quality Initiative. Cases were selected based on two distinct surgical sequences: (1) C-arm x-ray-assisted localization and marking of the puncture point, guide wire insertion, sufficient decompression and bone graft fusion, percutaneous screw placement and internal fixation. (2) C-arm x-ray-assisted localization and marking of the puncture point, sufficient decompression and bone graft fusion, guidewire insertion, percutaneous screw placement and internal fixation. In this study, such operations were consistently performed by the same senior surgeon at Hubei Provincial Hospital of Traditional Chinese Medicine from September 2019 to June 2023. We collected comprehensive data encompassing standard patient demographics, surgical details, clinical outcomes, complications, and imaging metrics specifically focusing on lumbar lordosis angle and intervertebral space height. Clinical outcomes were assessed quantitatively utilizing the Visual Analog Scale (VAS) scores and the Oswestry Disability Index (ODI) both preoperatively and at the one-year postoperative mark. A total of 143 patients met the inclusion and exclusion criteria and served as the final cohort for the present study. Among them, 84 patients underwent the surgical sequence of C-arm x-ray-assisted localization and marking of the puncture point, guide wire insertion, sufficient decompression and bone graft fusion, percutaneous screw placement and internal fixation (Group A). The remaining 59 patients underwent the sequence of C-arm x-ray-assisted localization and marking of the puncture point, sufficient decompression and bone graft fusion, guidewire insertion, percutaneous screw placement and internal fixation (Group B). All patients were followed for a minimum of 12 months, and All experiments using human subjects were performed in accordance with the Declaration of Helsinki and approved by the Ethics Committee of Hubei Provincial Hospital of Traditional Chinese Medicine.

### Inclusion and exclusion criteria

The inclusion criteria were as follows: (1) Clinical presentations were indicative of radicular symptoms associated with ULSS, such as neurogenic intermittent claudication; (2) Imaging criteria were indicative of ULSS, which included: (i) Lumbar instability was characterized by sagittal vertebral displacement exceeding 4 mm or a sagittal rotation angle greater than 10° as observed on dynamic lumbar radiographs (flexion-extension views); (ii) Lumbar spinal stenosis was defined by any form of narrowing within the spinal canal, nerve root canal, or intervertebral foramen; (3) The clinical manifestations and imaging findings of ULSS were consistent with the affected segments; (4) Standardized conservative treatments, including a minimum of six months of physical therapy, nonsteroidal anti-inflammatory drugs (NSAIDs), or epidural steroid injections, proved ineffective; (5) All patients underwent Endo-PLIF.

The exclusion criteria were as follows: (1) Lumbar degenerative spondylolisthesis was greater than Grade II; (2) History of spinal fracture, infection, or neoplasm was present; (3) Previous surgical procedures involving the lumbar spine were performed; (4) Significant comorbidities were present that could adversely influence surgical outcomes, including osteoarthritis, rheumatoid arthritis, or metabolic bone disorders; (5) Other severe medical conditions were present that contraindicated surgical intervention; (6) Follow-up period was less than one year or follow-up data were incomplete.

### Spinal endoscopic instruments

All procedures were conducted utilizing either the Endo-surgi Plus system or the Endo-surgi Standard system [Shanghai Maoyu Medical (Group) Co., LTD, China], with the optional use of an endoscopic high-speed bur or piezosurgery, contingent upon the specific surgical requirements. And the fusion procedure involved the use of a posterior lumbar fixation system and posterior lumbar interbody fusion cage (Fule Science & Technology Development Co., Ltd, Beijing, China).

### Surgical procedures

Group A: Upon the patient's entry into the operating room, an intravenous line was established, and continuous monitoring of electrocardiogram (ECG), peripheral oxygen saturation (SpO2), non-invasive blood pressure (NIBP), and bispectral index (BIS) was initiated. Following the induction of general anesthesia, patients were positioned prone.

Under C-arm x-ray fluoroscopy, the surface projection of the pedicle at the surgical segment was identified and marked. Routine preoperative disinfection and sterile draping of the surgical field were then performed.

Under C-arm x-ray machine fluoroscopy, the puncture needles were sequentially inserted at the bilateral pedicle entry points of the responsible segment. Subsequently, the inner core of each puncture needle was removed and guide wires were inserted through the puncture channels and secured externally. A 1.5 cm incision was made at the entry point on the decompression side, and sequential dilators were used to insert the working channel. Subsequently, the light source and endoscopic system were deployed.

A partial laminectomy, along with the partial resection of the superior and inferior articular processes, was executed utilizing a visualized trephine and rongeur. The excised bone was morselized and subsequently reinserted into the intervertebral space. The ligamentum flavum was adequately exposed and partially resected to facilitate visualization of the dural sac, nerve roots, and the annulus fibrosus of the intervertebral disc. Protruding and extraneous nucleus pulposus material was excised as required, and adhesions between the nerve roots, dural sac, and posterior longitudinal ligament were meticulously released. In cases of lateral recess stenosis, part of the superior lamina was removed to decompress the lateral recess until the nerve roots and dural sac were free of compression. For patients exhibiting bilateral lower limb symptoms, it is crucial to ensure thorough decompression of the contralateral spinal canal and lateral recess. Intraoperative confirmation of adequate decompression was achieved through direct endoscopic visualization based on the following criteria: (1) the absence of tension in the dural sac and the restoration of normal pulsation, (2) the unrestricted mobilization of both traversing and exiting nerve roots, ensuring no residual compression within the central canal, lateral recess, or neural foramen, and (3) the verification of sufficient space using a blunt probe. This procedure should be conducted until endoscopic assessment verifies the absence of substantial nerve root compression on both sides.

Endplate preparation instruments, such as curettes, were utilized to excise the inferior and superior endplates along with the intervertebral disc. Following adequate preparation of the intervertebral space, an appropriate quantity of autologous bone graft (composed of morselized bone harvested during the decompression phase, primarily from the resected lamina and facet joints) was implanted into the intervertebral space. Subsequently, an interbody fusion cage of suitable size was inserted and moderately expanded. The position and depth of the fusion cage were verified using fluoroscopy. Endoscopic inspection confirmed complete nerve root decompression on both the dorsal and ventral sides, with no significant bleeding observed.

In cases involving patients with two-segment disease, an identical surgical procedure was conducted on the other segment. This procedure similarly encompassed decompression, preparation of the intervertebral space, and insertion of an interbody fusion cage.

A transverse incision measuring 1.5 cm was made at the surface surrounding each guide wire site, facilitating the insertion of percutaneous pedicle screws and the installation of bilateral connecting rods, which were subsequently compressed as necessary. Fluoroscopic imaging was employed to verify the correct positioning of the screws within the pedicles, ensuring proper orientation and length, as well as to confirm the accurate placement of the connecting rods.

The surgical incisions underwent repeated irrigation with physiological saline, followed by the placement of a deep vacuum drain in each incision. The incisions were meticulously sutured in a layer-by-layer fashion, bandaged under sterile conditions, and secured. The operation was concluded.

The drains were extracted 24–48 h postoperatively. Patients were administered suitable anti-infective, analgesic, dehydration, and neurotrophic therapies as required. After drain removal, patients were advised to engage in moderate ambulation under the protection of the waist circumference and progressively perform exercises targeting the lumbar and back muscles. The patients were instructed to regularly recheck relevant examinations in the outpatient clinic and carry out various scores.

Group B: The anesthesia method and patient positioning were identical to Group A. The surgical procedure followed the sequence of C-arm x-ray-assisted localization and marking of the puncture point, sufficient decompression and bone graft fusion, guidewire insertion, percutaneous screw placement and internal fixation. Each specific step was identical to Group A. Incisions and postoperative management were also consistent with Group A.

### Data collection

The operative time, intraoperative fluoroscopy count, intraoperative bleeding, postoperative drainage volume, postoperative bed times for both groups were recorded. To evaluate the safety of the surgical procedure, intraoperative and postoperative complications were systematically documented, and the rates of these complications were subsequently calculated. Preoperative and one-year postoperative assessments included the Visual Analog Scale (VAS) scores and the Oswestry Disability Index (ODI). Preoperative and one-year postoperative radiographs were utilized to assess the lumbar lordosis angle, measure intervertebral space height at the surgical segment, and evaluate the status of interbody fusion. The excellent and good rate of pedicle screw placement was evaluated using postoperative CT scans based on the Gertzbein-Robbins criteria: Grade 0, screw entirely within the pedicle cortex; Grade I, screw breach of ≤2 mm without intraoperative neural or vascular complications; Grade II, screw breach of >2 mm without related complications; Grade III, complete cortical breach with associated neural or vascular complications. The excellent and good rate of pedicle screw placement was calculated as the number of Grade 0 and Grade I screws divided by the total number of screws placed (16). Definitive fusion was identified by the formation of trabecular bony bridges between contiguous vertebral bodies at the instrumented levels, intact hardware, and less than 3° segmental movement according to the fusion criteria (17).

### Statistical analysis

Normally distributed and homoscedastic quantitative data were expressed as mean ± standard deviation ($\bar{x}\pm s$). Data that did not conform to normal distribution were presented as median. For data conforming to normal distribution, intergroup comparisons between two groups were performed using independent samples t-tests, while comparisons among multiple groups were conducted using one-way ANOVA. Independent samples t-tests were employed for comparisons between groups. For data not conforming to normal distribution or homoscedasticity, significant difference in continuous variables were compared by the Mann–Whitney *U* test between the two groups and the Kruskal–Wallis *H* test among the three groups. Categorical data were expressed as rates or percentages and analyzed using chi-squared tests or Fisher exact tests. All figures were generated with the R software 4.2.1, *P* value < 0.05 were considered statistically significant.

## Results

### Comparison of basic demographic characteristics

A total of 143 eligible participants were enrolled in this study, comprising 84 individuals in Group A and 59 in Group B. Baseline demographic characteristics, including gender, age, body mass index (BMI), disease duration, and lesion segment, were evaluated and compared between the two groups. The analysis revealed no statistically significant differences between the groups (*P* > 0.05) (Table 1).

**Table 1:** Comparison of demographic characteristics in two groups.

Variable	Group A	Group B	*p* value
Age (year)	62.00 [48.75, 70.00]	57.00 [48.00, 69.50]	0.241
Gender			0.803
Female	40 (47.62%)	26 (44.07%)	
Male	44 (52.38%)	33 (55.93%)	
BMI (kg/m^2^)	21.50 [17.00, 26.00]	20.00 [16.00, 25.00]	0.392
Disease duration (year)	3.00 [2.00, 4.00]	3.00 [2.00, 4.00]	0.819
Lesion segment			0.834
L2/L3	3 (3.57%)	2 (3.39%)	
L3/L4	10 (11.90%)	10 (16.95%)	
L4/L5	36 (42.86%)	22 (37.29%)	
L5/S1	27 (32.14%)	22 (37.29%)	
L3/L4, L4/L5	1 (1.19%)	0 (0.00%)	
L4/L5, L5/S1	7 (8.33%)	3 (5.08%)	
Number of diseased segments			0.526
One-segment	76 (90.48%)	56 (94.92%)	
Two-segment	8 (9.52%)	3 (5.08%)	

BMI, body mass index.

### Comparison of surgical indicators

The operative time in Group A (169.50 [142.75, 196.50) was significantly shorter than in Group B (206.00 [192.00, 222.50), and the intraoperative fluoroscopy counts in Group A (13.00 [11.00, 16.00) were significantly lower than those in Group B (14.00 [12.00, 19.00) (*P* < 0.05). Additionally, there were no significant differences in postoperative drainage volume between Group A and Group B, nor in intraoperative bleeding and postoperative bedtimes between the two groups (*P* > 0.05). The complication rate in Group A was lower than in Group B (*P* > 0.05) (Table 2). In Group A, complications were observed in three instances: a dural tear accompanied by cerebrospinal fluid leakage, postoperative lower limb radicular sensory numbness, and persistent postoperative pain, resulting in a complication rate of 3.57%. In Group B, there were eight instances of surgical complications, comprising two cases of cerebrospinal fluid leakage, one case of suboptimal postoperative wound healing, two cases of postoperative lower limb radicular sensory numbness, and three cases of residual postoperative pain, resulting in a complication rate of 13.56%. The two cases of transient postoperative radicular sensory numbness and three cases of residual postoperative pain gradually improved with postoperative dehydration and neural nutrition therapy. The case of poor wound healing experienced delayed healing after enhanced wound dressing. No other complications were observed. There was no statistically significant difference in the incidence of surgical complications between the two groups (*P* > 0.05). In Group A, a total of 352 pedicle screws were implanted in 84 patients, comprising 297 Grade 0, 33 Grade I, and 20 Grade II screws, resulting in an excellent and good placement rate of 94.32%. Conversely, in Group B, 242 screws were implanted in 59 patients, with 157 Grade 0, 41 Grade I, and 44 Grade II screws, leading to an excellent and good placement rate of 81.82%. The rate of excellent and good pedicle screw placement in Group A was significantly higher than that in Group B.

**Table 2:** Comparison of surgery-related indicators between the two groups.

Variable	Group A	Group B	*p* value
Follow-up time (months)	15.00 [14.00, 17.00]	15.00 [14.00, 17.00]	0.897
Operative time (min)	169.50 [142.75, 196.50]	206.00 [192.00, 222.50]	<0.001
Intraoperative bleeding (ml)	52.50 [36.75, 65.00]	53.00 [41.00, 66.00]	0.686
Intraoperative fluoroscopy count	13.00 [11.00, 16.00]	14.00 [12.00, 19.00]	0.0017
Postoperative drainage volume (ml)	91.50 [54.75, 121.00]	100.00 [67.00, 121.50]	0.404
Postoperative bedtimes (days)	2.00 [1.00, 3.00]	2.00 [2.00, 3.00]	0.662
Postoperative complications			0.051
Non-occurrence	81 (96.43%)	51 (86.44%)	
Occurrence	3 (3.57%)	8 (13.56%)	

### Comparison of clinical and functional parameters

The follow-up period for both groups ranged from 12 to 24 months [15.00 (14.00, 17.00) months], with Group A at 15.00 [14.00, 17.00] months and Group B at 15.00 [14.00, 17.00] months. Statistical analysis indicated no significant difference between the two groups (*P* > 0.05). There were no significant differences in VAS and ODI scores between the two groups at baseline (*P* > 0.05). Postoperatively, both scores improved significantly over time, with statistically significant differences observed between the two groups (*P* < 0.05). In Group A, the VAS score decreased from 6.00 [5.00, 7.00] to 2.00 [1.00, 3.00], while in Group B it decreased from 6.00 [5.00, 6.00] to 2.00 [1.00, 3.00]. Furthermore, the ODI score in Group A decreased from 73.00 [65.00, 80.00] to 34.50 [28.00, 43.00], and in Group B from 77.00 [70.00, 85.50] to 37.00 [27.50, 44.00]. There were no significant differences between the groups at the same time points (*P* > 0.05) (Table 3).

**Table 3:** Comparison of clinical and functional parameters.

Variable	Group A	Group B	*p* value
Follow-up period (months)	15.00 [14.00, 17.00]	15.00 [14.00, 17.00]	0.897
VAS			
Preoperation	6.00 [5.00, 7.00]	6.00 [5.00, 6.00]	0.223
1 year postoperatively	2.00 [1.00, 3.00]	2.00 [1.00, 3.00]	0.362
*p* value	<0.05	<0.05	
ODI (%)			
Preoperation	73.00 [65.00, 80.00]	77.00 [70.00, 85.50]	0.010
1 year postoperatively	34.50 [28.00, 43.00]	37.00 [27.50, 44.00]	0.887
*p* value	<0.05	<0.05	

### Comparison of imaging data

No significant preoperative differences were observed between the groups in terms of lumbar lordosis angle and intervertebral space height at the surgical segment (*P* > 0.05). Postoperatively, both parameters showed significant improvement, which continued to increase over time, with statistically significant differences observed between the groups (*P* < 0.05). Comparisons at the same time points revealed no significant differences between the groups (*P* > 0.05). During the follow-up period, no displacement or breakage of instrumentation was observed, and there was no significant loss of intervertebral height. The fusion rate in both groups was 100% at one year postoperatively (Table 4).

**Table 4:** Comparison of imaging data in two groups.

Variable	Group A	Group B	*p* value
Lumbar lordosis angle (°)			
Preoperation	24.00 [19.00, 27.00]	23.00 [18.00, 27.00]	0.659
1 year postoperatively	33.00 [30.75, 37.00]	34.00 [30.00, 36.00]	0.471
*p* value	<0.05	<0.05	
Intervertebral space height (mm)			
Preoperation	4.00 [3.00, 6.00]	5.00 [4.00, 6.00]	0.104
1 year postoperatively	10.00 [8.00, 10.00]	10.00 [8.00, 10.00]	0.510
*p* value	<0.05	<0.05	

### Comparison of Odom criteria

At 1 year postoperatively, clinical outcomes in Group A, as assessed by Odom's criteria, were excellent in 51 cases (60.71%), good in 27 cases (32.14%), fair in 5 cases (5.95%), and poor in 1 case (1.19%). In Group B, outcomes were excellent in 37 cases (62.71%), good in 15 cases (25.42%), fair in 4 cases (6.78%), and poor in 3 cases (5.08%). The overall rate of excellent or good clinical outcomes was 92.85% in Group A and 88.13% in Group B, with no statistically significant difference between the groups (*P* > 0.05) (Table 5).

**Table 5:** Comparison of Odom criteria between the two groups.

Variable	Criteria	Group A	Group B	*p* value
Odom				0.506
Excellent	Improvement of preoperative symptoms and signs	51 (60.71%)	37 (62.71%)	
Good	Improvement but limitation of activity	27 (32.14%)	15 (25.42%)	
Fair	Partial relief of symptoms with full activity	5 (5.95%)	4 (6.78%)	
Poor	Symptoms and signs unchanged or exacerbated	1 (1.19%)	3 (5.08%)	

### Comparison of typical cases

**Figure 1:**
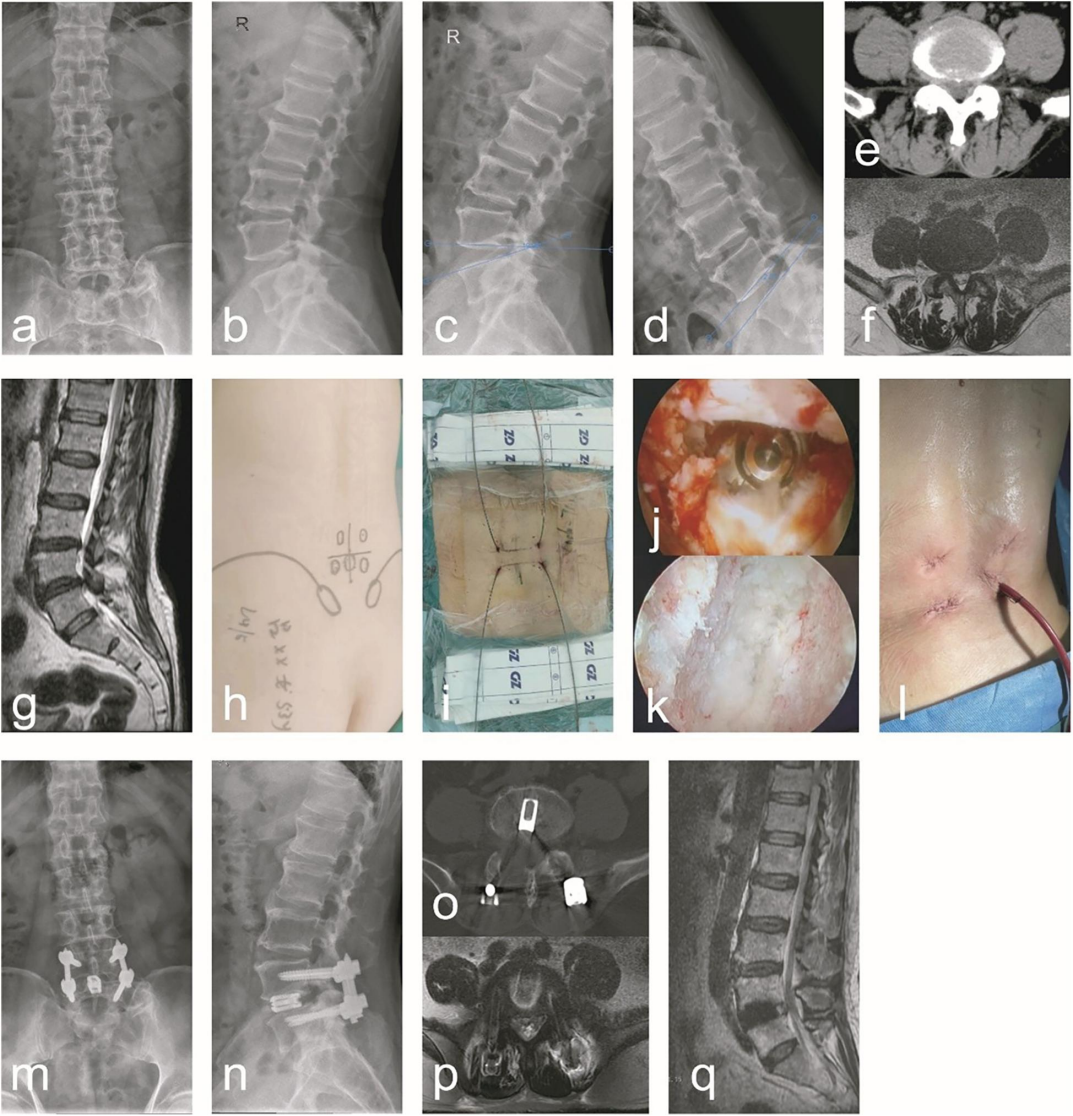
A typical case of C-arm x-ray-assisted localization and marking of the puncture point, guide wire insertion, sufficient decompression and bone graft fusion, percutaneous screw placement and internal fixation (Group A). A 67-year-old male patient had radiating low back pain with numbness and weakness in the right lower limb. **(a–g)** Preoperative anteroposterior and lateral lumbar x-rays, hyperextension and hyperflexion x-rays, CT, and MRI revealed lumbar instability and L4/5 lumbar spinal stenosis. Additionally, ligamentum flavum hypertrophy, facet joint osteophytes, disc herniation with calcification, and right nerve root compression were observed. **(h)** During the surgical procedure, the affected pedicle segment was identified and marked using C-arm x-ray fluoroscopic guidance. **(i)** During surgery, guidewires were preferentially inserted prior to decompression. **(j)** After addressing the responsible intervertebral segment intraoperatively, endoscopic examination of the superior and inferior endplates confirmed thorough treatment. **(k)** Following the insertion of the interbody fusion cage intraoperatively, endoscopic visualization confirmed adequate decompression and re-expansion of the nerve root, with the fusion cage positioned correctly. **(l)** A general picture of the wound after operation. **(m–q)** Postoperative follow-up x-ray, CT, and MRI demonstrated proper positioning of the pedicle screws and fusion cage, adequate interbody bone grafting, and unobstructed cerebrospinal fluid signals at the L4/5 segment. Notable improvements were observed in spinal canal stenosis, lumbar instability, and nerve root compression at this segment.

Group A: A male, 67 years old, was admit ted due to “low back pain with numbness and weakness in the right lower limb for 20 years”. Diagnosis: ULSS, degenerative L4/5 disc herniation. The typical case data are shown in Figure 1.

**Figure 2:**
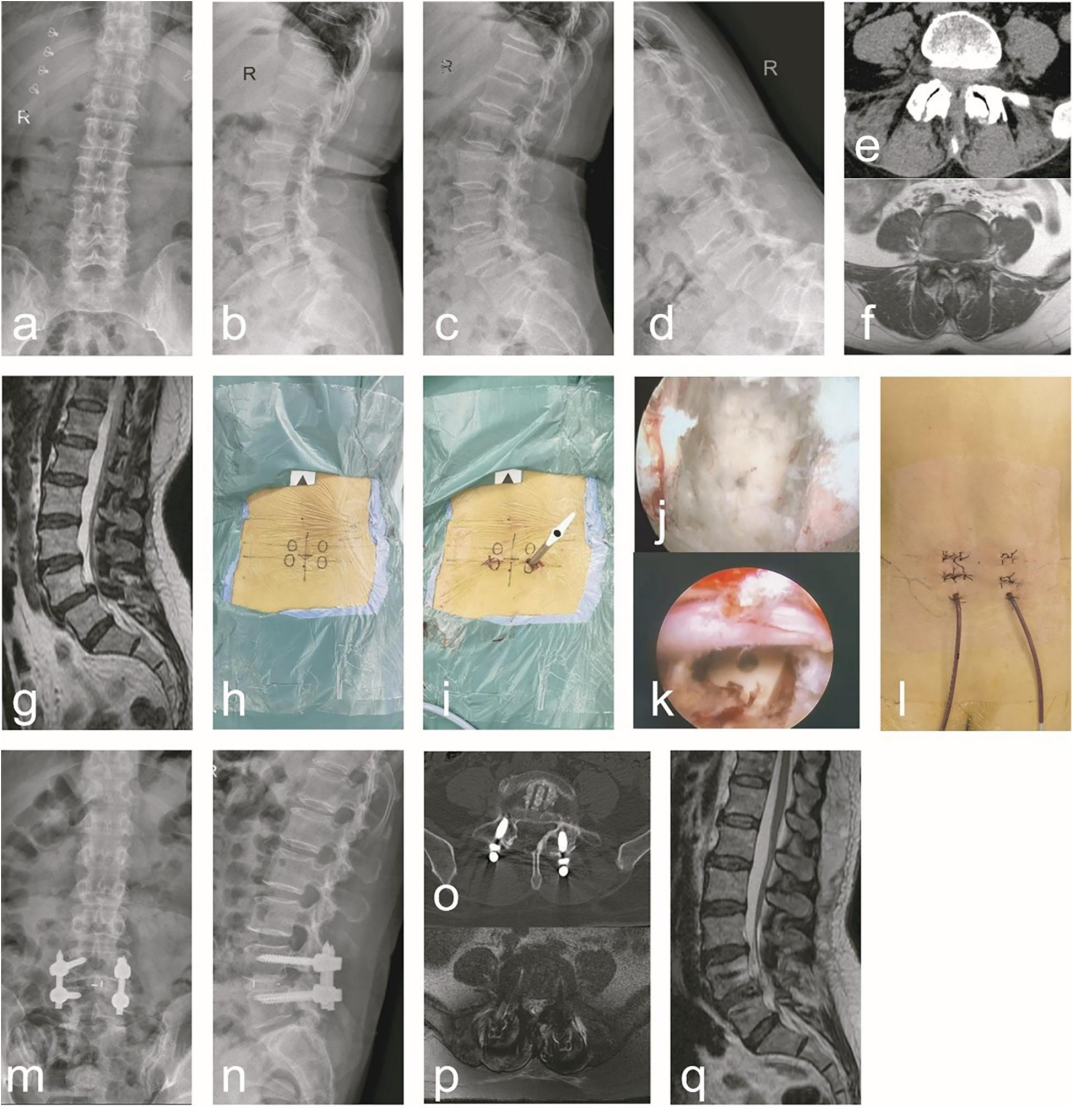
A typical case of C-arm x-ray-assisted localization and marking of the puncture point, sufficient decompression and bone graft fusion, guidewire insertion, percutaneous screw placement and internal fixation (group B). A 56-year-old female patient had radiating low back pain with numbness and weakness in the right lower limb. **(a–g)** Preoperative anteroposterior and lateral lumbar x-rays, hyperextension and hyperflexion x-rays, CT, and MRI revealed lumbar instability and L4/5 lumbar spinal stenosis. Additionally, ligamentum flavum hypertrophy, facet joint osteophytes, disc herniation, and right nerve root compression were observed. **(h)** During the surgical procedure, the affected pedicle segment was identified and marked using C-arm x-ray fluoroscopic guidance. **(i)** Intraoperatively, endoscopic decompression was performed first. Following decompression, guidewires were inserted, and subsequent steps were carried out. **(j)** After addressing the responsible intervertebral segment intraoperatively, endoscopic examination of the superior and inferior endplates confirmed thorough treatment. **(k)** Following the insertion of the interbody fusion cage intraoperatively, endoscopic visualization confirmed adequate decompression and re-expansion of the nerve root, with the fusion cage positioned correctly. **(l)** A general picture of the wound after operation. **(m–q)** Postoperative follow-up x-ray, CT, and MRI demonstrated proper positioning of the pedicle screws and fusion cage, adequate interbody bone grafting, and unobstructed cerebrospinal fluid signals at the L4/5 segment. Notable improvements were observed in spinal canal stenosis, lumbar instability, and nerve root compression at this segment.

Group B: A female, 56 years old, was admit ted to the hospital due to “low back pain with numbness and weakness in the right lower limb for 1 years, aggravated for 1 week”. Diagnosis: ULSS, degenerative L4/5 disc herniation. The data are shown in Figure 2.

## Discussion

This retrospective study sought to evaluate the efficacy of two distinct surgical sequences, designated as Group A and Group B, in the context of Endo-PLIF for managing ULSS. Both groups attained satisfactory clinical outcomes; nonetheless, Group A exhibited reduced operative times, decreased intraoperative fluoroscopy count, and a superior rate of excellent and good pedicle screw placement. At present, there is a notable paucity of literature comparing the clinical outcomes of these two surgical sequences in Endo-PLIF. Consequently, this study aimed to compare the perioperative data and clinical outcomes associated with the two surgical sequences in the management of ULSS, utilizing Endo-PLIF techniques. Initial findings indicate that Group A represents the more favorable surgical sequence for this procedure.

Endoscopic lumbar interbody fusion was initially introduced by Leu in 1996 (18). Nonetheless, the procedure did not gain widespread adoption at that time due to technological limitations and insufficient development of supporting instruments (19, 20). With the ongoing advancements in endoscopic lumbar decompression and fusion techniques, coupled with the progressive development of endoscopic surgical instruments and tools, endoscopic surgery has once again captured the interest of spinal surgeons (21–23). Compared to earlier research findings, recent studies indicate that the efficacy and safety of endoscopic lumbar interbody fusion have significantly improved (24). However, the steep learning curve, prolonged operative time, and higher incidence of complications associated with endoscopic lumbar interbody fusion have significantly limited its widespread application (25, 26). Optimizing surgical sequence and reducing operative time are among the key research directions for further promoting this technique. Despite the advantages of minimally invasive surgery, Endo-PLIF remains technically demanding. Our findings suggest that early guide wire insertion (Group A) not only improves surgical efficiency but also enhances screw placement accuracy, likely due to more consistent anatomical landmarks prior to decompression. These results align with prior reports emphasizing the importance of workflow optimization in minimally invasive spine surgery (27, 28).

The Endo-PLIF procedure can be categorized into two distinct phases. The first phase encompasses decompression, interbody bone grafting, and fusion, whereas the second phase entails puncture, guidewire insertion, and the placement of pedicle screws using the guidewire. It is important to note that the phases are executed sequentially, with decompression, bone grafting, and fusion occurring prior to the puncture, guidewire insertion, and subsequent percutaneous placement of pedicle screws. Theoretically, this sequence exhibits greater coherence and aligns more effectively with the operational practices associated with the percutaneous pedicle screw placement phase; however, the early insertion of the guidewire may disrupt the decompression process. Despite this, clinical practice often adheres to the sequence of C-arm x-ray-assisted localization and marking of the puncture point, followed by adequate decompression and bone graft fusion, guidewire insertion, percutaneous screw placement, and internal fixation for endoscopic lumbar fusion. Nevertheless, given that decompression entails the excision of portions of the lamina and facet joints, conducting decompression prior to guidewire insertion may alter anatomical landmarks, thereby complicating the localization of the puncture site. Furthermore, decompression diminishes the dimensions of the superior articular process and partially removes the lamina, which elevates the risk of the puncture needle inadvertently breaching the spinal canal. This, in turn, increases the complexity and duration of screw placement. Consequently, the sequential process involving C-arm x-ray-assisted localization and marking of the puncture point, guide wire insertion, sufficient decompression and bone graft fusion, percutaneous screw placement and internal fixation represents a more optimal approach for endoscopic lumbar fusion. Additionally, securing the guide wire to the body surface with sterile adhesive film post-insertion can mitigate interference during the decompression procedure.

To date, there is a notable lack of literature comparing the clinical outcomes associated with these two sequences of Endo-PLIF. This study conducted a retrospective analysis of data from 143 patients diagnosed with ULSS, all of whom underwent Endo-PLIF performed by our minimally invasive spine surgery team between October 2019 and June 2023. The primary objective was to compare the clinical outcomes associated with the two surgical sequences previously discussed. The results revealed significant differences in operative time and intraoperative fluoroscopy count between the two groups, with Group A demonstrating more favorable metrics than Group B. However, the study found no significant differences in perioperative indicators, including intraoperative bleeding and postoperative drainage volume. These results suggest that the initial placement of the guidewire contributes to a reduction in intraoperative radiation exposure and a decrease in operative time, thereby minimizing the potential cumulative impact of radiation on both patients and the surgical team. thereby minimizing the potential cumulative impact of radiation on both patients and the surgical team. In contrast, conducting decompression prior to guidewire insertion appears to increase the complexity and duration of screw placement. At the one-year postoperative interval, both cohorts demonstrated substantial enhancements in Visual Analog Scale (VAS) scores and Oswestry Disability Index (ODI) relative to preoperative values, with no statistically significant differences observed between the groups. This suggests comparable efficacy of the two surgical sequences. Furthermore, both groups exhibited significant improvements in lumbar lordosis angle and intervertebral space height at the 1-year postoperative mark, highlighting the effectiveness of both surgical approaches in restoring physiological spinal curvature and intervertebral space height. These factors are critical for achieving favorable medium- and long-term outcomes (29). The lack of significant differences between the groups indicates that the sequence of surgical procedures does not substantially influence the restoration of physiological spinal curvature and intervertebral space height. The notable decrease in operative time and radiation exposure associated with Sequence A (early guidewire placement) holds considerable clinical significance. Reduced procedure durations mitigate anesthetic risks for patients, enhance operating room turnover efficiency, and diminish cumulative radiation exposure for the surgical team, thereby addressing critical practical concerns in contemporary spine surgery. Furthermore, the higher rate of excellent and good outcomes in pedicle screw placement observed in Group A underscores the benefit of guidewire insertion prior to decompression, facilitating more precise screw placement.

No intraoperative complications such as nerve injury, spinal cord injury, incomplete decompression, or injury to the prevertebral vascular structures occurred in either group, indicating that both surgical sequences are safe and feasible for treating ULSS. However, this study identified two instances of dural tears accompanied by cerebrospinal fluid leakage, which were likely attributable to severe adhesion of the dural sac to surrounding tissues resulting from an extended duration of the disease. To mitigate the risk of complications, it is recommended that surgeons exercise meticulous and gentle handling of tissues during procedures, particularly when excising nucleus pulposus tissue. Additionally, precise control and accurate assessment of the depth of the nucleus pulposus forceps are imperative to prevent injury to the dural sac. In this study, five patients exhibited postoperative regional numbness in the lower limbs, which ameliorated following symptomatic treatment focused on nerve nourishment and edema reduction. Five patients experienced residual postoperative pain, likely attributable to injury to the nerve root sheath or edema of the nerve root, which subsequently resolved following dehydration and nerve-nourishing interventions. Additionally, one patient exhibited poor wound healing postoperatively, a condition linked to a history of diabetes and inadequately controlled perioperative blood glucose levels. The wound ultimately achieved delayed healing through the implementation of more frequent dressing changes.

While endoscopic surgery is characterized by its minimally invasive nature and smaller incisions, it is imperative to effectively manage patients' underlying conditions during the perioperative period and to actively mitigate factors that may hinder wound healing (30). Prior research has demonstrated a significant positive correlation between operative duration and the occurrence of surgical complications (31, 32). No significant differences were found in fusion rates, overall complication rates, or final clinical outcome scores (VAS, ODI) between groups at one year, indicating that both sequences are fundamentally safe and effective for achieving the primary goals of ULSS surgery. It is important to acknowledge that the complication rate observed in Group A (3.57%) was numerically lower compared to Group B (13.56%). Although this difference did not achieve statistical significance (*p* = 0.051), it indicates a potentially clinically relevant trend, suggesting that the “guidewire-first” sequence may offer a safety advantage. The main limitations of this study include its retrospective design, single-center setting, relatively modest sample size, and short to mid-term follow-up period. The absence of randomization inherently raises the potential for selection bias, as unmeasured variables, including nuanced anatomical variations or the surgeon's preferences on a given day, may have influenced the sequence of treatment selection. Conducting a prospective randomized controlled trial would yield the highest level of evidence to conclusively validate our findings. Furthermore, the findings represent the outcomes attained by a specialized team in minimally invasive spine surgery at a single institution, utilizing a technique unique to a senior surgeon. The extent of efficiency improvements and the reduction in complication rates may differ when implemented by surgeons with varying levels of expertise or in different clinical environments. Future prospective, multicenter studies with larger cohorts and longer follow-up are needed to confirm these findings and assess the long-term durability of the outcomes.

In conclusion, both surgical sequences for Endo-PLIF in the treatment of ULSS are both feasible and effective.

## Conclusion

The clinical outcomes of two sequences of Endo-PLIF for patients with ULSS are similar. However, the sequence involving C-arm x-ray-assisted localization and marking of the puncture point, guide wire insertion, sufficient decompression and bone graft fusion, percutaneous screw placement and internal fixation, results in shorter operative time, fewer fluoroscopy count, and higher excellent and good rate of pedicle screw placement. While the selection of the surgical sequence is ultimately influenced by a range of patient-specific factors and the preferences of the surgeon, this particular sequence will offer a more promising approach for Endo-PLIF.

## Data Availability

The raw data supporting the conclusions of this article will be made available by the authors, without undue reservation.

## References

[1] Deyo RA. Treatment of lumbar spinal stenosis: a balancing act. Spine J. (2010) 10(7):625–7. 10.1016/j.spinee.2010.05.006 20620984

[2] Deyo RA Mirza SK Martin BI. Back pain prevalence and visit rates: estimates from U.S. national surveys, 2002. Spine. (2006) 31(23):2724–7. 10.1097/01.brs.0000244618.06877.cd 17077742

[3] Deyo RA Mirza SK Martin BI Kreuter W Goodman DC Jarvik JG. Trends, major medical complications, and charges associated with surgery for lumbar spinal stenosis in older adults. JAMA. (2010) 303(13):1259–65. 10.1001/jama.2010.338 20371784 PMC2885954

[4] Taylor WR Chen JW Meltzer H Gennarelli TA Kelbch C Knowlton S Quantitative pupillometry, a new technology: normative data and preliminary observations in patients with acute head injury. Technical note. J Neurosurg. (2003) 98(1):205–13. 10.3171/jns.2003.98.1.0205 12546375

[5] Katz JN Zimmerman ZE Mass H Makhni MC. Diagnosis and management of lumbar spinal stenosis: a review. JAMA. (2022) 327(17):1688–99. 10.1001/jama.2022.5921 35503342

[6] Zaina F Tomkins-Lane C Carragee E Negrini S. Surgical versus non-surgical treatment for lumbar spinal stenosis. Cochrane Database Syst Rev. (2016) 2016(1):CD010264. 10.1002/14651858.CD010264.pub2 26824399 PMC6669253

[7] Chu EC Sabourdy E. Non-surgical restoration of L3/L4 disc herniation. Cureus. (2023) 15(6):e40941. 10.7759/cureus.40941 37496528 PMC10368486

[8] Lee SH Erken HY Bae J. Corrigendum to “percutaneous transforaminal endoscopic lumbar interbody fusion: clinical and radiological results of mean 46-month follow-up”. BioMed Res Int. (2017) 2017:3431257. 10.1155/2017/3431257 28785578 PMC5529624

[9] Osman SG. Endoscopic transforaminal decompression, interbody fusion, and percutaneous pedicle screw implantation of the lumbar spine: a case series report. Int J Spine Surg. (2012) 6:157–66. 10.1016/j.ijsp.2012.04.001 25694885 PMC4300894

[10] Wagner R Haefner M. Uniportal endoscopic lumbar interbody fusion. Neurospine. (2020) 17(Suppl 1):S120–8. 10.14245/ns.2040130.065 32746525 PMC7410390

[11] Wang L Li C Han K Chen Y Qi L Liu X. Comparison of clinical outcomes and muscle invasiveness between unilateral biportal endoscopic discectomy and percutaneous endoscopic interlaminar discectomy for lumbar disc herniation at L5/S1 level. Orthop Surg. (2023) 15(3):695–703. 10.1111/os.13627 36597673 PMC9977580

[12] Isaacs RE Podichetty VK Santiago P Sandhu FA Spears J Kelly K Minimally invasive microendoscopy-assisted transforaminal lumbar interbody fusion with instrumentation. J Neurosurg Spine. (2005) 3(2):98–105. 10.3171/spi.2005.3.2.0098 16370298

[13] Mummaneni PV. Percutaneous transforaminal lumbar interbody fusion for the treatment of degenerative lumbar instability. Neurosurgery. (2008) 62(6):E1384. 10.1227/01.neu.0000333326.57953.e0 18824969

[14] Schizas C Tzinieris N Tsiridis E Kosmopoulos V. Minimally invasive versus open transforaminal lumbar interbody fusion: evaluating initial experience. Int Orthop. (2009) 33(6):1683–8. 10.1007/s00264-008-0687-8 19023571 PMC2899194

[15] Hagan MJ Remacle T Leary OP Feler J Shaaya E Ali R Navigation techniques in endoscopic spine surgery. BioMed Res Int, (2022) 2022:8419739. 10.1155/2022/8419739 36072476 PMC9444441

[16] Gertzbein SD Robbins SE. Accuracy of pedicular screw placement *in vivo*. Spine. (1990) 15(1):11–4. 10.1097/00007632-199001000-00004 2326693

[17] Schwender JD Holly LT Rouben DP Foley KT. Minimally invasive transforaminal lumbar interbody fusion (TLIF): technical feasibility and initial results. J Spinal Disord Tech. (2005) 18:S1–6. 10.1097/01.bsd.0000132291.50455.d0 15699793

[18] Leu HF Hauser RK. Percutaneous endoscopic lumbar spine fusion. Neurosurg Clin N Am. (1996) 7(1):107–17. 10.1016/S1042-3680(18)30410-8 8835151

[19] Nagahama K Ito M Abe Y Murota E Hiratsuka S Takahata M. Early clinical results of percutaneous endoscopic transforaminal lumbar interbody fusion: a new modified technique for treating degenerative lumbar spondylolisthesis. Spine Surg Relat Res. (2018) 3(4):327–34. 10.22603/ssrr.2018-0058 31768452 PMC6834458

[20] Wu J Liu H Ao S Zheng W Li C Li H Percutaneous endoscopic lumbar interbody fusion: technical note and preliminary clinical experience with 2-year follow-up. BioMed Res Int. (2018) 2018:5806037. 10.1155/2018/5806037 30581859 PMC6276503

[21] Momin AA Steinmetz MP. Evolution of minimally invasive lumbar spine surgery. World Neurosurg. (2020) 140:622–6. 10.1016/j.wneu.2020.05.071 32434014

[22] Hussain I Hofstetter CP Wang MY. Innovations in spinal endoscopy. World Neurosurg. (2022) 160:138–48. 10.1016/j.wneu.2021.11.099 35364672

[23] Tan R Lv X Wu P Li Y Dai Y Jiang B Learning curve and initial outcomes of full-endoscopic posterior lumbar interbody fusion. Front Surg. (2022) 9:890689. 10.3389/fsurg.2022.890689 35574552 PMC9096087

[24] Relvas-Silva M Pinto BS Sousa A Loureiro M Pinho AR Pereira P. Is endoscopic technique an effective and safe alternative for lumbar interbody fusion? A systematic review and meta-analysis. EFORT Open Rev. (2024) 9(6):536–55. 10.1530/EOR-23-0167 38828975 PMC11195334

[25] Brusko GD Wang MY. Endoscopic lumbar interbody fusion. Neurosurg Clin N Am. (2020) 31(1):17–24. 10.1016/j.nec.2019.08.002 31739925

[26] Jacquot F Gastambide D. Percutaneous endoscopic transforaminal lumbar interbody fusion: is it worth it? Int Orthop. (2013) 37(8):1507–10. 10.1007/s00264-013-1905-6 23657674 PMC3728384

[27] Ishii K Watanabe G Tomita T Nikaido T Hikata T Shinohara A Minimally invasive spinal treatment (MIST)-a new concept in the treatment of spinal diseases: a narrative review. Medicina (Kaunas, Lithuania). (2022) 58(8):1123. 10.3390/medicina58081123 36013590 PMC9413482

[28] Vaishnav AS Othman YA Virk SS Gang CH Qureshi SA. Current state of minimally invasive spine surgery. J Spine Surg (Hong Kong). (2019) 5(Suppl 1):S2–10. 10.21037/jss.2019.05.02 PMC6626758 31380487

[29] Goldstein CL Macwan K Sundararajan K Rampersaud YR. Perioperative outcomes and adverse events of minimally invasive versus open posterior lumbar fusion: meta-analysis and systematic review. J Neurosurg Spine. (2016) 24(3):416–27. 10.3171/2015.2.SPINE14973 26565767

[30] Senker W Stefanits H Gmeiner M Trutschnig W Weinfurter I Gruber A. Does obesity affect perioperative and postoperative morbidity and complication rates after minimal access spinal technologies in surgery for lumbar degenerative disc disease. World Neurosurg. (2018) 111:e374–85. 10.1016/j.wneu.2017.12.075 29274450

[31] Jenkins NW Parrish JM Hrynewycz NM Brundage TS Singh K. Complications following minimally invasive transforaminal lumbar interbody fusion: incidence, independent risk factors, and clinical impact. Clin Spine Surg. (2020) 33(5):E236–40. 10.1097/BSD.0000000000000933 31913178

[32] Sousa JM Ribeiro H Silva JL Nogueira P Consciência JG. Clinical outcomes, complications and fusion rates in endoscopic assisted intraforaminal lumbar interbody fusion (iLIF) versus minimally invasive transforaminal lumbar interbody fusion (MI-TLIF): systematic review and meta-analysis. Sci Rep. (2022) 12(1):2101. 10.1038/s41598-022-05988-0 35136081 PMC8825843

